# The Clinical Profile of Patients with COPD Is Conditioned by Age

**DOI:** 10.3390/jcm12247595

**Published:** 2023-12-09

**Authors:** Diego Morena, José Luis Izquierdo, Juan Rodríguez, Jesús Cuesta, María Benavent, Alejandro Perralejo, José Miguel Rodríguez

**Affiliations:** 1 Pulmonology Department, Respiratory Medicine, Hospital Universitario de Guadalajara, 19002 Guadalajara, Spain; jlizquierdoa@gmail.com; 2 Doctoral Program in Health Sciences, University of Alcalá, 28871 Alcalá de Henares, Spain; 3 Department of Medicine and Medical Specialties, University of Alcalá, 28871 Alcalá de Henares, Spain; jesuscuestadomingo@gmail.com (J.C.); respirama@yahoo.es (J.M.R.); 4 Geriatric Medicine, Hospital Universitario de Guadalajara, 19002 Guadalajara, Spain; jrodriguezs@sescam.jccm.es; 5 SAVANA, 28013 Madrid, Spain; mbenavent@savanamed.com (M.B.); aparralejo@savanamed.com (A.P.); 6 Respiratory Medicine, Hospital Universitario Príncipe de Asturias, 28805 Alcalá de Henares, Spain

**Keywords:** COPD, big data, artificial intelligence, age, comorbidities, daily clinical practice

## Abstract

In recent years, many studies have analyzed the importance of integrating time, or aging, into the equation that relates genetics and the environment to the development and origin of COPD. Under conditions of daily clinical practice, our study attempts to identify the differences in the clinical profile of patients with COPD according to age and the impact on the global burden of the disease. This study is non-interventional and observational, using artificial intelligence and data captured from electronic medical records. The study population included patients who were diagnosed with COPD between 2011 and 2021. A total of 73,901 patients had a diagnosis of COPD. The mean age was 73 years (95% CI: 72.9–73.1), and 56,763 were men (76.8%). We observed a specific prevalence of obesity, heart failure, depression, and hiatal hernia in women (*p* < 0.001), and ischemic heart disease and obstructive sleep apnea (OSA) in men (*p* < 0.001). In the analysis by age ranges, a progressive increase in cardiovascular risk factors was observed with age. In conclusion, in a real-life setting, COPD is a disease that primarily affects older subjects and frequently presents with comorbidities that are decisive in the evolutionary course of the disease.

## 1. Introduction

Chronic obstructive pulmonary disease (COPD) is one of the leading causes of morbidity and mortality worldwide. Symptoms usually appear after the age of 40, although the clinical presentation varies greatly at the onset of the disease, during its progression, and in terms of its clinical impact at different stages of life. This variability requires an understanding of all the factors that determine the burden of the disease in any given patient [1,2]. The current pharmacological treatment of COPD is based on the results of large clinical trials that select subjects who represent the average patient, but they do not always analyze the associated diseases that influence the clinical deterioration of these patients. Furthermore, the main clinical trials on which the evidence for the pharmacological treatment of COPD is based is usually restricted to a mean patient age of 65 + 8 years [3,4,5,6,7]. In many cases, this patient selection prevents the results from being adequately extrapolated to a large number of patients who may require personalized treatment due to their specific characteristics.

In order to correctly understand what happens in patients with COPD, we must consider that the patient’s clinical situation is often conditioned by other diseases that are not always related to the degree of limitation observed on spirometry and which also increasingly progress with age [8,9]. In fact, a common finding in patients with COPD is that, even with similar forced expiratory volume in 1 s (FEV1) levels, we may observe different patterns of functional impairment, dissimilar clinical manifestations, a variable number of exacerbations, and varying degrees of quality of life. Part of this variability may be related to the heterogeneity of the disease itself, which possibly originates in different pathogenic mechanisms and is reflected in the clinical phenotypes. However, several studies carried out in the general population have shown that, as the age of patients with COPD increases, it is common for more than one chronic comorbidity to appear, which contributes towards the accentuated clinical deterioration of these patients [10,11].

In addition to presenting a greater number of comorbidities, age itself has been described as having a potential influence on the progression of COPD. Nonetheless, although similarities have been described between lung aging (within the concept of “senile lung”) and COPD, the physiological changes that occur with age cannot be interpreted as a pathological process that requires intervention. There is currently not enough evidence to establish whether lung aging alone is highly relevant in patients with COPD who require medical care or hospitalization for exacerbation; in fact, it would only explain certain associated pathological changes—mainly emphysema [12]. Regardless of the role that aging may have in the pathogenesis of COPD, what is indisputable is that, as they age, patients with COPD present different clinical characteristics that must be identified, as they can decisively influence disease progression.

In recent years, many studies have analyzed the importance of integrating time, or aging, into the equation that relates genetics and the environment to the development and origin of COPD. The age of an individual when the interaction between genetics and the environment occurs is important, as well as the previous exposures suffered by said individual, or even their parents. A genetic–environmental–temporal approach will provide not only more information on lung functionality, but also determine the heterogeneity of clinical presentation in COPD [13].

Our hypothesis is that the clinical profile of COPD varies with patient age, resulting in differences in the burden of the disease. This factor should be considered when designing clinical studies and proposing specific treatment programs, thereby making it possible to plan more personalized patient management focused on the main treatable traits, while potentially reducing the current burden of COPD.

The application of big data techniques and artificial intelligence in healthcare enable us to manage and extract value from complex data generated in large volumes from electronic health records (EHR). Thanks to this technology, it is possible to evaluate the main indicators of a certain clinical process, avoiding selection biases beyond the very existence of the registry. Big data has emerged as a fundamental tool in the current era, transforming the way we approach and understand complex phenomena in various fields, including epidemiology. Its utility in the contemporary age is particularly highlighted in the field of public health, where the massive collection and analysis of data have enabled the early detection of diseases, the monitoring of epidemiological patterns, and the formulation of more effective preventive strategies.

In the context of epidemiology, big data facilitates the integration of data from various sources, such as electronic health records, social media data, and wearable health devices. Additionally, big data analysis allows for more precise customization of health interventions and policies, tailoring them to the specific needs of different communities. Moreover, the marriage of big data and artificial intelligence (AI) further enhances our capabilities for health management. AI plays a crucial role in processing and analyzing large datasets generated by big data. Advanced algorithms can identify patterns and correlations in real time, enabling early disease detection and a faster response to critical epidemiological situations. The machine learning capability of AI also contributes to the continuous improvement of predictive models, refining their accuracy over time as new data is incorporated. In this regard, the synergy between big data and AI emerges as a fundamental pillar to strengthen epidemiological resilience and promote global health in the present day.

The objective of this study is to identify, under conditions of normal clinical practice, the differences in the clinical profile of patients with COPD according to age and their impact on the global burden of the disease. In this way, we can obtain a better understanding of these gene–environmental–temporal interactions in order to provide higher quality treatments and prevention of this disease.

## 2. Materials and Methods

We designed an observational, retrospective and non-interventional study using secondary data captured from the free text of electronic medical records (EMR). This study was conducted in the Castilla-La Mancha region of Spain, whose healthcare administration (SESCAM) utilizes the Savana Manager v.3.0 tool, making it possible to analyze data going back to 1 January 2011. In doing so, we have followed the Strengthening the Reporting of Observational Studies in Epidemiology (STROBE) guidelines [14]. The study population included all patients over the age of 40 with a diagnosis of COPD who had been treated between 1 January 2011 and 24 January 2021.

The methodology we used has been described in previous publications [15,16,17,18]. Savana Manager is a data extraction system based on artificial intelligence (natural language processing, NLP) and big data techniques. This technology is able to extract unstructured clinical information (natural language or free text) from electronic history records (EHR) and transform it into reusable and organized information for research purposes, while maintaining the anonymous nature of patients at all times. In addition, through the use of computational linguistic techniques, comprehensive clinical content is scientifically detected and validated by the SNOMED CT [19] tool using data from the EHR of the SESCAM specialized care network (hospitalization, emergency, and outpatient consultations) and primary care services.

Data management and protection: The hospital IT services are responsible for processing the data and rendering it anonymous so that Savana subsequently receives non-identifiable data. In addition, an algorithm is used during data extraction to randomly insert confounding information per patient, while at the same time, only recovering part of the individual information. The end result of this methodology is the creation of a completely dissociated and anonymous patient database, so that all study reports contain only aggregated data, and neither patients nor physicians can be identified. According to the European Data Protection Authority, once an anonymous clinical record is cleared of personal data, the General Data Protection Regulation no longer applies. This study was approved by the Research Ethics Committee (Comité de Ética de la Investigación, or CEIm) of the Guadalajara public healthcare area (ref 1/2023, date 17 January 2023).

Information extraction assessment: For the scope of this study, variable COPD was detected in the free text using a named-entity recognition approach. As additional layers, negation and temporality detection were applied. The machine learning model to detect negation is a combination of a rule-based layer with a binary convolutional neural network trained on real Spanish EHRs and evaluated against a rich set of reference standards. This model classifies each clinical entity as being affirmative or non-affirmative, based on its lexical and semantic context. The temporality detection is carried out by an NLP module that consists of various layers that work in combination to assign dates to clinical entities. The first layer is a named-entity detection engine responsible for the detection of any mentioning of dates in the free text of the EHRs. Subsequently, a relationship model based on a Bi-LSTM decides if a detected date is related to a detected clinical entity. In addition, a normalization layer converts different date formats, as written in the EHRs’ free text, into a normalized representation. The last step in NLP processing, the post-processing step, carries out several quality control operations and combines the output from the different NLP modules into a final database.

After the cNLP processing, three authors validated the results of the tool and the performance of the technology. The purpose of this evaluation was to verify the validity of the EHRead^®^ technology in the identification of records containing mentions of “COPD” and related variables. A set of 560 documents was manually verified, which ensured the reliability of the manual annotation/review and constituted the gold standard. The performance of Savana was calculated using a gold standard evaluation resource created by the experts, i.e., the accuracy of Savana in identifying records in which the presence of the disease under study, and the related variables detected, were measured with respect to the gold standard. The performance was calculated by the standard metrics of precision (P), recall (R), and the F-score, which is the harmonic mean of the two previous metrics.

Precision indicated the reliability of the information retrieved by the system and was calculated as P = tp/(tp + fp). Recall, an indicator of the amount of information retrieved by the system, was calculated as R = tp/(tp + fn). The F-score was calculated as F = 2 × precision × recall/(precision + recall). This parameter provided an indicator of overall information retrieval performance. In all cases, true positives (tp) were the sum of correctly identified records, false negatives (fn) were the sum of unidentified records, and false positives (fp) were the sum of incorrectly retrieved records.

We have previously described that the values for these metrics were greater than 0.9, indicating that the diagnosis was adequate for identifying the study population. The F values of the different terms included in the analyses ranged between 0.92 and 0.97.

Statistical analysis: All variables were evaluated using SPSS software (version 25.0; IBM, Armonk, NY, USA) and OpenEpi (https://www.OpenEpi.com accessed on 6 February 2023). We used standard descriptive statistical analyses. Qualitative variables are expressed as absolute frequencies and percentages, while quantitative variables are expressed as means, 95% confidence intervals, and standard deviations. For the analysis of numerical variables, the Student’s *t*-test for independent measures was used, while the Chi-squared test was used to measure the association and compare proportions between qualitative variables. To assess whether the variables analyzed were related with the population selected, significance was assessed using a Chi-squared 2 × 2 contingency table, controlling for sex and age biases. A *p*-value less than 0.05 was considered statistically significant. Savana presents the events in order, according to the odds ratio (observed vs. expected frequency). In all cases, differences whose *p*-value associated with the contrast test was less than 0.05 were considered significant.

## 3. Results

**Figure 1:**
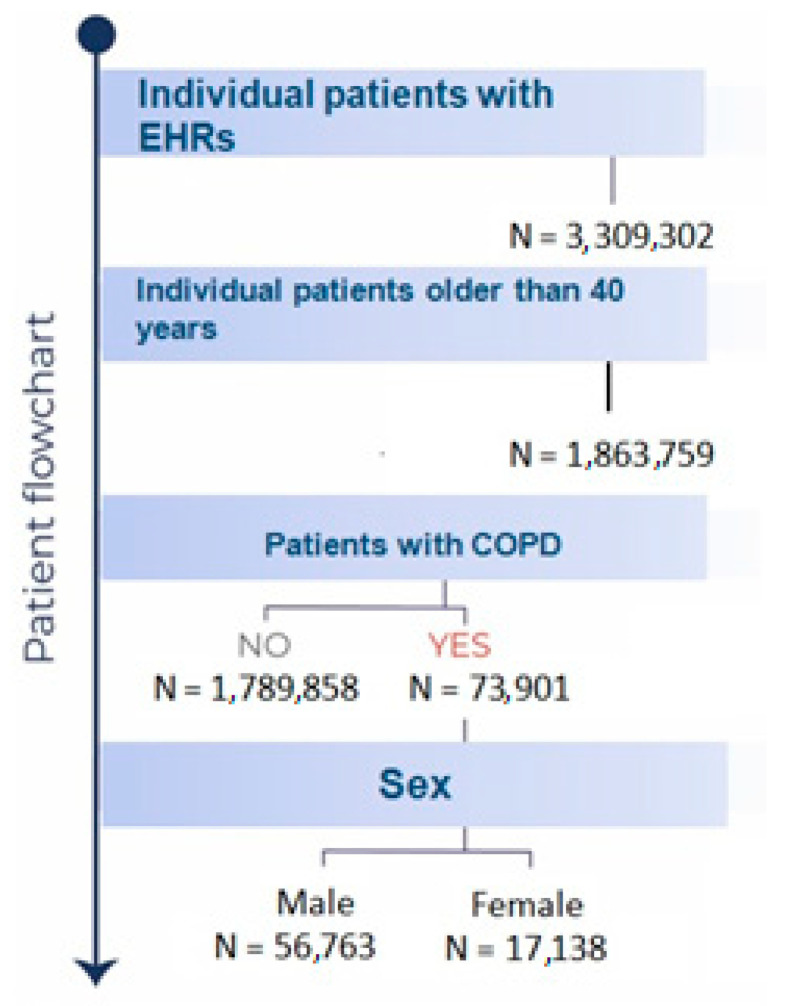
Flowchart of patients included in this study.

During the study period (1 January 2011 to 14 January 2021), 73,901 patients diagnosed with COPD were treated by Castilla-La Mancha Public Healthcare Services (SESCAM). The mean age was 73 years (95% CI: 72.9–73.1), and 76.8% of patients were male (56,763). Figure 1 presents the flowchart of the patients included in this study.

The main clinical and demographic characteristics of the study population are shown in Table 1.

When we analyzed the patients by sex, we found that obesity, heart failure, depression, and hiatal hernia were especially prevalent in women (*p* < 0.001), while ischemic heart disease and obstructive sleep apnea (OSA) were more prevalent in men (*p* < 0.001).

In the analysis by age ranges, a progressive increase in cardiovascular risk factors is observed with age. In addition, a progressive increase in associated diseases, especially cardiovascular diseases, is also observed (Table 2).

In fact, in patients over the age of 70, heart failure is present in a high percentage of patients (30.9% from 70–79 years of age, and 58.7% over 80 years of age). Compared to the general 40+ population, patients with COPD have a statistically significantly higher incidence of cardiovascular risk factors, cardiovascular disease, depression, and hiatal hernia.

Table 3 shows the impact of age on the burden of disease, measured by hospital admissions and mortality.

This factor is relevant since the percentage of hospital admissions and mortality increases significantly, but it also represents a very high percentage of the entire COPD population.

**Figure 2:**
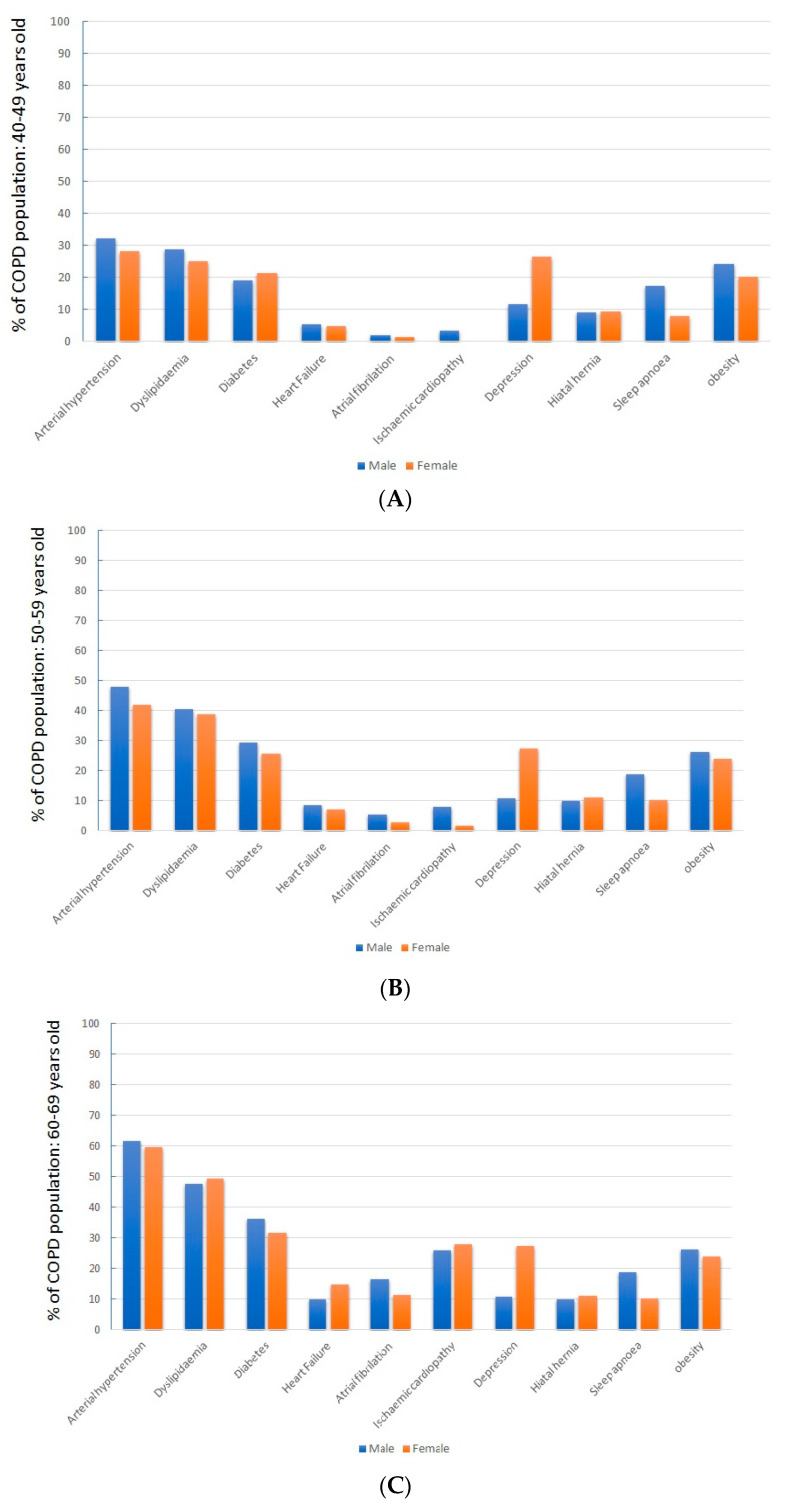

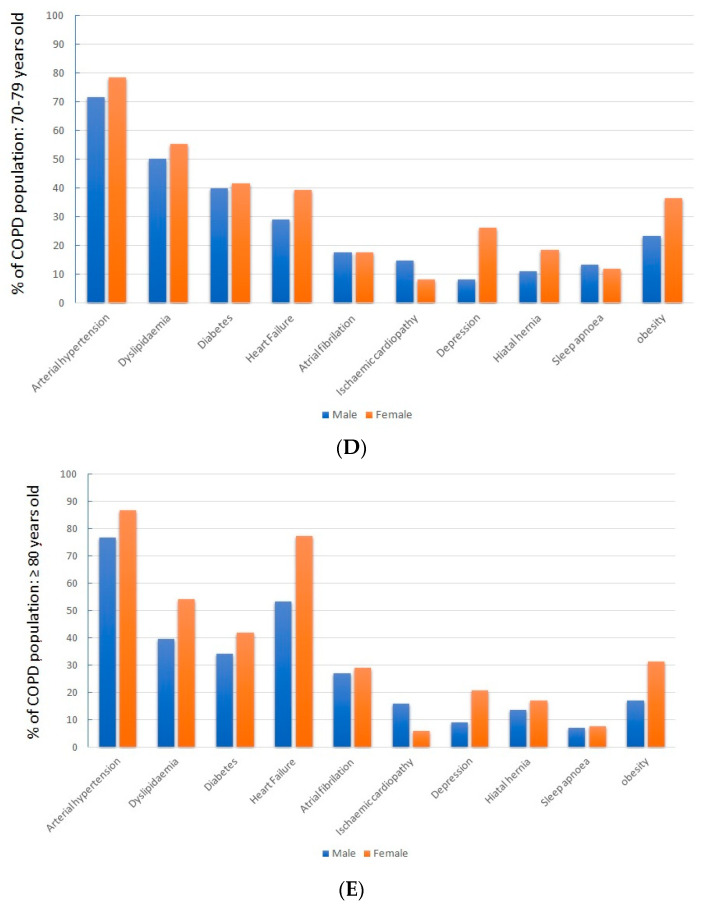
Differences in comorbidities by sex in different age ranges above 40 years. (**A**) Population between 40–49 years; (**B**) population between 50–59 years; (**C**) population between 60–69 years; (**D**) population between 70–79 years; (**E**) population over 80 years.

The sex-related differences observed in the general population were maintained over all age ranges (Figure 2).

## 4. Discussion

The data from this study confirm that most patients with COPD have an associated complexity of conditions, which partly correlates with comorbidities and increases progressively with age. As these patients age, changes in their profile are associated with a greater consumption of healthcare resources (hospitalizations) and mortality.

COPD should not be seen as a single disease, but as a syndrome that encompasses functional and structural alterations, causing recognizable chronic symptoms. This is due to the interactions between the environment and genetics, but with time (aging) as a direct collaborator. The final biological and clinical results that is produced by the genetic-environmental interactions and those previous accumulated interactions—in regards to the patient, as well as to their parents—present a crucial axis, which is time, or aging [13]. Knowledge about the usefulness of this factor in COPD could allow for the identification of new early therapeutic and preventive targets for this disease.

Certain associated comorbidities (such as cardiovascular disease, especially heart failure) can have a decisive effect on the clinical symptoms of patients. Using data from the ECLIPSE study, Agustí et al. proposed a shared pathogenic mechanism [20]. However, there are no data to confirm either a causal relationship or a mere association motivated by a greater coincidence of risk factors. In any event, as clearly demonstrated by our study, patients with COPD have a high prevalence of comorbidities, which can be decisive in the clinical expression of COPD, some of which are significantly associated with increased morbidity and mortality [21]. Several observational studies have previously described the higher prevalence of comorbidities in patients with COPD compared to the general population, and this is also noted in our setting [8,22]. However, the most important achievement of this study is that it quantifies the importance of the problem in a real-life setting, without the selection biases of most previous observational studies, identifying its age-adjusted magnitude.

Several studies have shown an inverse correlation between the patient’s health status and the existence of comorbidities, especially when three or more are associated, regardless of lung function [23,24,25]. Additionally, the risk of exacerbation and hospitalization, mortality, and the economic impact of the disease on healthcare systems are related to the number of comorbidities [26,27,28].

Some series have shown that, compared to the general population, patients with COPD studied under real-life conditions have a higher frequency of obesity, depression, obstructive sleep apnea (OSA), and hiatal hernias [29,30,31], whose frequency does not intensify with the age of the patient. However, other comorbidities may have a greater impact, depending on patient age. For instance, this age-related increase is highly significant in cardiovascular diseases. Heart failure is common among patients with COPD, but it can be decisive in certain patients over the age of 70, since its presence can not only deteriorate the baseline clinical situation of the patient, but also simulate or aggravate exacerbations.

Based on our data, which are current and obtained from a real-life setting, we can confirm that COPD is a disease that predominantly affects elderly patients, and that this age group has a higher concentration of patients, as well as cases of more complex disease, causing a greater impact on healthcare services. The use of big data methods and artificial intelligence has allowed us to obtain a realistic overview of the treatment needs of patients in a given region, indicating that more than 50% of patients with COPD in our setting were over 70 years of age. These data are consistent with the results of the EPISCAN2 study, which has recently demonstrated the prevalence of COPD in Spain [32]. In this study, only 6.03% of COPD patients were 40 to 49 years of age, while 30.08% were 70 to 79 years of age. However, a particularly important group of patients, which is the over-80 age group, was not analyzed. In our series, this group represents 36% of the total.

One limitation of this study is that it included patients whose diagnosis of COPD had not always been confirmed with post-bronchodilator spirometry. This is especially relevant in patients over the age of 80. However, as they are diagnosed and treated as COPD patients, the great importance of this population in terms of treatment requirements is evident. As most previous studies do not include this population, it seems reasonable to assess specific strategies for these age groups, since only individualized treatment that considers each patient’s particularities will allow us to correctly manage these patients.

Multimorbidity, or comorbidity, are aspects of complexity and may be factors that do not fully explain the patient’s situation. Some patients with a single disease may require complex management, while others may present with many diseases, but may be easy to manage. For this reason, the assessment of comorbidity cannot replace functional evaluation as a means of obtaining a diagnosis, prognostic markers, or therapeutic objectives, and even less so in a type of population in which the specific weight of each disease is diluted under the overall impact of the accumulation of multiple alterations in various physiological systems. Despite that, the most remarkable and novel study presented here is the use of the big data approach. We believe that it can end the long-term permanent exclusion of the elderly from studies and models of chronicity and multimorbidity, despite being the population group with the highest prevalence of pathologies and for which health expenditures are concentrated. They are the real patients of our health system [33,34].

## 5. Conclusions

The main conclusion of this study, whose strength is that it has included a large population in a real-life setting, is that COPD is currently a disease that primarily affects patients of advanced age who frequently present with comorbidities that are decisive in the evolutionary course of the disease. A comprehensive understanding of the patient, and not merely of the disease alone, will be crucial for the correct management of most patients with COPD, and the use of frailty assessment instruments developed by geriatric specialists will allow for a better understanding of this group of patients and therefore, the application of measures tailored to their needs. Meanwhile, the most advanced age group comprises the greatest disease burden, both for the patient, as well as the public healthcare system. Knowledge about the usefulness of the time factor in COPD (GETomicis [13]) could allow for the identification of new early therapeutic and preventive targets for this disease, adding to the previously determined genetic and environmental factors.

## Figures and Tables

**Table 1:** Basal characteristics of the study population.

	Male COPD Population (n = 56,763)	Female COPD Population (n = 17,138)	*p*
Age, years (95% CI)	72.9 (72.8–73)	72.3 (72.1–72.5)	<0.001
**Comorbidities**			
Arterial hypertension (%)	70.0	72.3	<0.001
Dyslipidemia (%)	49.6	52.5	<0.001
Diabetes (%)	37.9	38.5	<0.001
Smoking (%)	41.7	35.9	<0.001
Obesity (%)	23.9	32.7	<0.001
Heart failure (%)	37.3	48.3	<0.001
Atrial fibrillation (%)	19.4	18.4	<0.01
Ischemic cardiopathy	14.4	7.7	<0.001
Obstructive sleep apnea (%)	13.5	10.8	<0.001
Depression (%)	10.0	27.2	<0.001
Hiatal hernia (%)	12.7	17.3	<0.001

**Table 2:** Comorbidities in COPD by age.

Age Range, Years Mean (CI 95%)	>40 without COPD 62.1 (62–62.1)	Total COPD Population > 40 73 (72.9–73.1)	*p*	COPD 40–49 45 (44.9–45.1)	COPD 50–59 54 (54.7–54.8)	COPD 60–69 64 (64.3–64.4)	COPD 70–79 74.4 (74.4–74.5)	>COPD 80 85.3 (85.3–85.4)
Sex, male (%)	51.4	76.8	<0.001	66.7	71.2	79.4	82.1	77.1
**Comorbidities**								
Arterial hypertension (%)	29.9	70.5	<0.001	30.8	46.2	61.1	72.7	78.7
Dyslipidemia (%)	21.0	50.3	<0.001	27.4	39.9	47.8	51.0	47.2
Diabetes (%)	14.4	38.1	<0.001	19.6	28.2	35.3	40.0	37.2
Smoking (%)	11.2	40.3	<0.001	70.3	68.2	52.0	32.1	17.7
Obesity (%)	8.2	25.9	<0.001	22.8	25.5	26.2	25.7	20.4
Heart failure (%)	6.9	40.1	<0.001	5.1	8.0	10.6	30.9	58.7
Atrial fibrillation (%)	4.5	19.1	<0.001	1.9	4.5	9.2	17.4	27.5
Ischemic cardiopathy	2.7	12.9	<0.001	2.6	6.4	10.3	13.5	14.7
Obstructive sleep apnea (%)	2.5	12.9	<0.001	14.1	16.3	15.4	11.0	7.1
Depression (%)	6.9	14.0	<0.001	16.4	15.5	12.0	11.2	11.7
Hiatal hernia (%)	5.1	13.8	<0.001	9.1	10.2	10.8	12.4	14.3

**Table 3:** Burden of the disease evaluated by hospitalizations and hospital mortality.

Age Range, Years	40–49	50–59	60–69	70–79	>80
% of total COPD population	3.2	11.9	20.8	28.0	36.1
% COPD patients that required hospitalization for acute deterioration	11.9	14.7	19.9	24.2	30.7
Number of hospitalizations among patients that required hospitalization	2.3	2.5	2.9	3.0	3.0
In-hospital death	3.7	2.6	3.2	4.3	6.2

## Data Availability

The data presented in this study are available upon request from the corresponding author. The data are not publicly available because they belong to the National Health System of Castilla La Mancha.
